# Guillain-Barré Syndrome: A Sequela of the Original COVID-19 Infection or Vaccination

**DOI:** 10.7759/cureus.28044

**Published:** 2022-08-15

**Authors:** Usman Ilyas, Zaryab Umar, Rubal Bhangal, Deesha Shah, Barry Fayman

**Affiliations:** 1 Internal Medicine, Icahn School of Medicine at Mount Sinai, Queens Hospital Center, Jamaica, USA

**Keywords:** guillain-barré syndrome, covid-19, intravenous immunoglobulin (ivig), covid-19 infection, therapeutic plasmapheresis, covid-19 vaccination

## Abstract

Since the beginning of the coronavirus disease 2019 (COVID-19) pandemic, many studies have reported the association between Guillain-Barré syndrome (GBS), COVID-19 infection, and vaccination. We present a case of a 62-year-old male who, six days after receiving the Pfizer-BioNTech COVID-19 vaccination, presented with acute unilateral limb weakness progressing to flaccid quadriparesis and decreased deep tendon reflexes. Of note, he also had four days of diarrhea before receiving the COVID-19 booster shot and tested positive for COVID-19 upon admission. He received five days of intravenous immunoglobulin (IVIG) followed by five cycles of plasmapheresis with minimal improvement in his neurological motor symptoms. Subsequently, he was discharged to an acute in-patient physical rehabilitation facility to improve his strength and mobility further. This case report sheds light on one of the neurological manifestations associated with the current COVID-19 pandemic, which may arise from either the viral infection, vaccination, or both.

## Introduction

As of July 30, 2022, 581,434,211 cases of coronavirus disease 2019 (COVID-19) have been reported worldwide. Pulmonary manifestations are the most commonly reported and range from mild flu-like symptoms to severe illnesses, including pneumonia, acute respiratory distress syndrome (ARDS), and respiratory failure. Furthermore, the involvement of the central (CNS) and peripheral (PNS) nervous systems has been widely documented [1,2]. The neurological manifestations can vary from headaches, anosmia, and dizziness to more severe findings, including cerebrovascular insults such as stroke, encephalitis, and encephalopathy [2]. In addition, studies have reported the association between Guillain-Barré syndrome (GBS), COVID-19, and vaccination [3-6]. The possible underlying mechanisms include direct damage caused by neuroinvasive properties of the virus, immune-mediated inflammatory response, and antibody-mediated damage. For the diagnosis of GBS, cerebrospinal fluid (CSF) examination demonstrating albuminocytologic dissociation (ACD) and electrodiagnostic studies is required [7]. Treatment options remain the same, including intravenous immunoglobulin (IVIG), plasma exchange, and aggressive physical therapy [8]. We describe the case of a 62-year-old male patient who presented with acute-onset unilateral limb weakness, which later progressed to progressive flaccid quadriparesis six days after receiving the COVID-19 vaccination. Upon presentation to the emergency department, the patient tested positive for COVID-19 infection. The clinical and laboratory evidence led to the diagnosis of GBS.

## Case presentation

**Figure 1:**
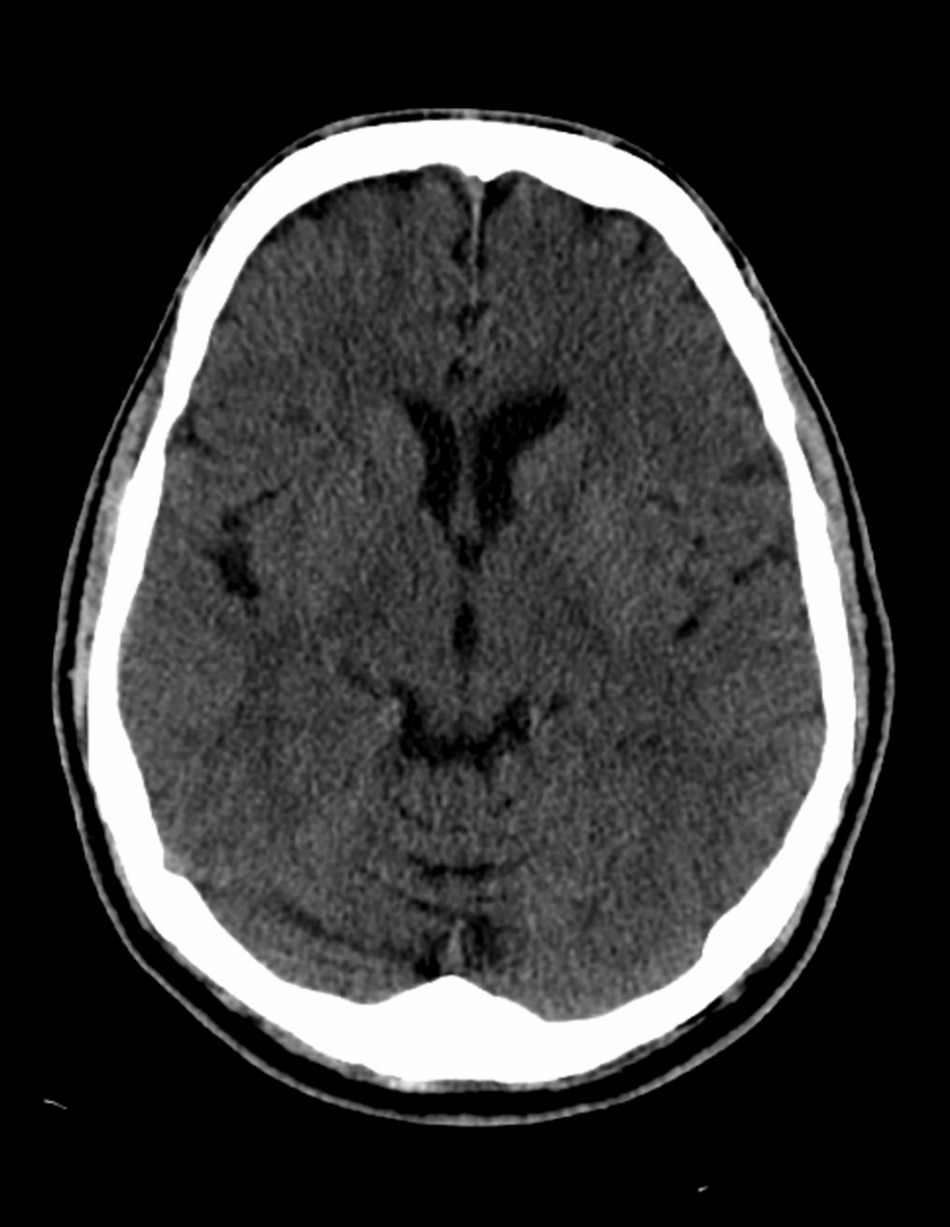
CT of the head without contrast showing no acute intracranial pathology.

**Figure 2:**
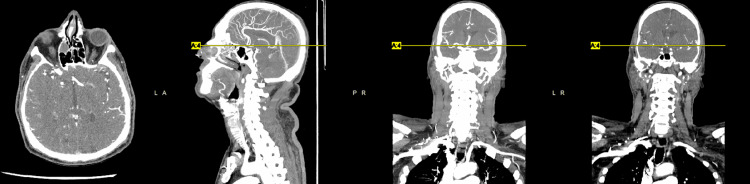
CT angiography of the brain showing no acute large vessel occlusion.

**Figure 3:**
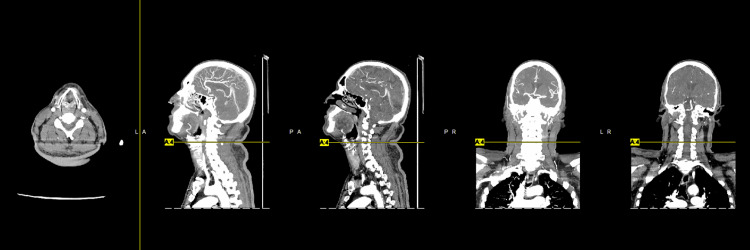
CT angiography of the neck demonstrating no hemodynamically significant carotid artery stenosis.

We present the case of a 62-year-old male with a past medical history of diabetes mellitus, hypertension, hyperlipidemia, and coronary artery disease requiring coronary artery bypass grafting in 2009 complicated by cerebral vascular disease with no residual weakness who presented to the emergency department with generalized weakness, most significant in his right hand of one-day duration. He also had subjective fever and chills. The patient stated that his symptoms began six days ago, the same day he received the COVID-19 vaccine booster. He had already received two doses of the Pfizer vaccine seven months ago. Of note, immediately before receiving the COVID-19 booster vaccination, the patient had four days of diarrhea. He denied slurred speech, chest pain, shortness of breath, nausea, vomiting, diarrhea, recent travel, and sick contact. Physical examination was relatively benign, except for 3/5 strength in both upper and lower extremities. Vitals, basic metabolic panel, and complete blood count were within normal limits. However, he tested positive for COVID-19 upon admission. He did not exhibit any respiratory symptoms and did not require any supplemental oxygen. Head computed tomography (CT) showed no acute intracranial pathology (Figure 1). CT angiogram of the brain (Figure 2) and neck (Figure 3) were negative for acute vessel occlusion.

The patient’s weakness quickly worsened as his strength decreased to 1/5 in both upper and lower extremities with decreased deep tendon reflexes and developed mild dysphagia. Neurology was consulted and recommended performing a lumbar puncture followed by starting IVIG therapy for possible GBS. CSF findings (Table 1) were significant for mildly elevated protein of 58 mg/dL (normal range: 15-45 mg/dL) and normal white blood cell (WBC) count of 2/mcL (normal range: 0-5/mcL). These findings demonstrate ACD that is typical of GBS.

**Table 1:** Cerebrospinal fluid (CSF) analysis findings consistent with albuminocytologic dissociation

Laboratory parameter	Reference range and unit	Value
Color	Colorless	Colorless
Appearance	Clear	Clear
WBC manual	0-5/mcL	3
WBC Ct	0-5/mcL	2
RBC manual	0-1/UL	5
RBC Ct	0-0/mcL	5
Protein	15-45 mg/dL	58
Glucose	40-70 mg/dL	66
Oligoclonal banding	Absent	Absent
IGG	≤3.4 mg/dL	4.7
Albumin	14-25 mg/dL	40.2
Acid-fast		No acid-fast bacilli isolated after six weeks
Culture		No growth at 14 days

The patient received five days of IVIG at a rate of 400 mg/kg and five cycles of plasmapheresis, after which he showed minimal improvement in his generalized weakness. Due to his worsening dysphagia, the patient developed aspiration pneumonia and required 10 days of 3 g ampicillin-sulbactam every six hours. Following this, a speech-language pathologist evaluated and recommended that the patient would benefit from a percutaneous endoscopic gastrostomy (PEG) tube placement as he continued to have a high risk of recurrent aspiration. He received nutritional feeding after the placement of a PEG tube. After an evaluation for a possible tracheostomy, the otolaryngology team recommended that it was not indicated at this time. On hospital day 28, the patient finally tested negative for COVID-19 and was discharged to an acute in-patient physical rehabilitation facility to improve his strength and mobility further.

## Discussion

As of July 30, 2022, a total of 581,434,211 cases of COVID-19 have occurred worldwide. Since the first case of COVID-19 in Wuhan, China, in December 2019, it was thought that COVID-19 was principally a respiratory illness [9]. Pulmonary manifestations constitute the most common presentation varying from mild symptoms such as fever, cough, and sore throat to severe illnesses involving but not limited to pneumonia, ARDS, and respiratory failure [9]. Initially, COVID-19 was thought to be a respiratory illness, but as more cases came to light, COVID-19 is an established multiorgan system disease, with varying atypical presentations of COVID-19 infection known [10,11].

Neurological involvement, both CNS and PNS, of COVID-19 has been documented widely [1,2]. Presentations vary from headache, anosmia, and dizziness to more severe consequences, including thromboembolic complications such as ischemic stroke, GBS, encephalitis, and encephalopathy [2]. Early in the pandemic, one such retrospective study done among hospitalized patients in Wuhan, China, showed that 36.4% of all hospitalized patients had some form of nervous system manifestation: CNS, PNS, and skeletal muscle [12]. The most common reported CNS manifestations were headache and dizziness, which occurred in 16.8% and 13.1%, respectively, while PNS manifestation involved smell and taste impairment, which occurred in 5.1% and 5.6%, respectively [12]. More severe neurological manifestation occurred in patients with “severe infection.” Cerebrovascular accidents occurred in 5.7%, impaired consciousness in 14.8%, and skeletal muscle injury in 19.3% [12]. In another systematic review, stroke was reported as one of the most common forms of nervous system manifestation (53.9%), followed by GBS (15.1%), meningitis, encephalitis, and encephalopathy (6.8%) [13].

Studies have reported the association between GBS and COVID-19 infection and vaccination [3-6]. GBS is an immune-mediated inflammatory polyradiculoneuropathy typically presenting as an ascending weakness/paralysis associated with hypo/areflexia [14]. In addition to weakness, patients might also have sensory symptoms, autonomic dysfunction, and ataxia [14]. Approximately 65% of cases are associated with a preceding infection, 4-6 weeks before the onset of symptoms [15]. *Campylobacter jejuni* is, to date, the most commonly linked organism [15]. Others include *Mycoplasma pneumoniae*, *Cytomegalovirus*, *Hepatitis E virus*, Epstein-Barr virus, and *Zika virus* [15]. Other less commonly linked preceding events include immune checkpoint inhibitor therapy, ganglioside administration, and surgery [15]. In the past, some cases were attributed to immunization. One such outbreak was in 1976, during the swine influenza vaccination campaign, where a 7.3-fold increase in the risk of GBS was noted [15,16].

The most commonly presenting preceding symptoms include fever, cough, sore throat, and diarrhea [17]. A retrospective study in Japan showed that fever was the most common preceding symptom (52%), while diarrhea occurred in 27% of those diagnosed with GBS and its variants [17,18]. GBS typically presents as an ascending weakness, but varying presentations have been reported worldwide. Those associated with COVID-19 infection also have reported atypical descending paralysis starting with facial muscle involvement [3,7]. Our patient reported diarrhea that started five days before the onset of unilateral upper extremity muscle weakness, which later involved all limb muscles. One systematic review showed that the symptoms of GBS start about 5-24 days after the onset of COVID-19 infection. In our case, whether the patient was asymptomatic at the onset of GBS symptoms or if symptoms of both GBS and COVID-19 started together is unclear [3]. Although a diagnosis of GBS can be made clinically, confirmation requires CSF examination and electrodiagnostic studies [7]. ACD, a normal cell count with elevated protein count on CSF examination, is classical for diagnosing GBS; however, 30% of patients in the first week of the disease are found to have a normal CSF protein count [7]. Our patient had the classic ACD finding on CSF examination. Other laboratory testing and imaging (such as brain MRI) can aid in excluding other causes of acute flaccid paralysis. In addition, a positive anti-ganglioside antibody level can aid diagnosis when in doubt, but a negative test result does not exclude the diagnosis [15]. Treatment options remain the same, including IVIG, plasma exchange, and aggressive physical therapy [8]. Our patient received five cycles of IVIG and plasma exchange before being discharged to a long-term rehabilitation facility.

Although the pathogenesis of GBS itself is unclear, it has been attributed to molecular mimicry and antibody-mediated neurological injury, specifically anti-ganglioside antibodies [19]. Various pathological mechanisms have been attributed to the causation of GBS in COVID-19-infected patients: direct damage caused by the neuroinvasive property of the virus, immune-mediated inflammatory response, and antibody-mediated injury [19]. A systematic review of 82 such cases found that only six cases had positive anti-ganglioside antibodies, and most had elevated inflammatory markers at the beginning of the neurological symptoms; thus, the cell-mediated immune response was hypothesized as the likely cause [19].

In another study, none of the patients had a positive CSF polymerase chain reaction test for severe acute respiratory syndrome coronavirus 2, pointing toward the immune-mediated mechanism [6]. Therefore, whether the peripheral nerve damage occurs due to molecular mimicry or T-cell-mediated inflammatory response is unclear [6]. Further studies investigating this matter are needed.

## Conclusions

This case report sheds light on the neurological manifestations associated with the current COVID-19 pandemic, possibly due to the viral infection itself or a consequence of the COVID-19 vaccination. Apart from stroke, meningitis, encephalitis, and encephalopathy, GBS should also be on the differentials when handling patients with signs and symptoms suggestive of a neurological etiology. The case report also highlights the approach to diagnosing and managing patients presenting with features suggestive of GBS. We hope that the discussion will help readers understand the possible pathophysiology associated between GBS and COVID-19 infection and vaccination. However, more research is required to understand better the disease in this era of the COVID-19 pandemic and to aid in early diagnosis and better management.
